# A brachialis muscle rupture diagnosed by ultrasound; case report

**DOI:** 10.1186/1865-1380-4-46

**Published:** 2011-07-26

**Authors:** Titus JA Schönberger, Miranda F Ernst

**Affiliations:** 1 Emergency Department, Jeroen Bosch Hospital, 's-Hertogenbosch, The Netherlands; 2 Department of Surgery, Jeroen Bosch Hospital, 's-Hertogenbosch, The Netherlands

## Abstract

Trauma to the elbow caused by lifting heavy objects frequently involves rupture of the tendon of the biceps brachii muscle. Less frequently a rupture of the brachialis muscle occurs. To our knowledge, only five cases involving traumatic rupture of the brachialis muscle were described in the past 20 years. We will briefly report these cases.

To demonstrate and evaluate muscle injuries, magnetic resonance imaging (MRI) is considered the most sensitive and specific method of choice. We report an isolated brachialis muscle rupture caused by resisted flexion and pronation of the lower arm. Physical examination combined with ultrasound evaluation confirmed the diagnosis of ruptured brachialis muscle. Treatment was non-operative with full restoration of function.

## Background

Trauma to the elbow caused by lifting heavy objects frequently involves rupture of the tendon of the biceps brachii muscle. Less frequently a rupture of the brachialis muscle occurs. After an extensive online search, we found only five cases involving traumatic rupture of the brachialis muscle had been described in the past 20 years. To demonstrate and evaluate muscle injuries, magnetic resonance imaging (MRI) is considered the most sensitive and specific method of choice. We report an isolated brachialis muscle rupture caused by resisted flexion and pronation of the lower arm. Physical examination combined with ultrasound evaluation confirmed the diagnosis of ruptured brachialis muscle. Treatment was non-operative.

## Case presentation

A 45-year-old male, right-handed, amateur bodybuilder and metalworker presented to our emergency department with pain in the left elbow after lifting his motorcycle. At the time of injury, he noticed a sudden snap in his left elbow and felt immediate pain and weakness. There were no previous injuries to the elbow, but the patient reported a visible dell on the medial surface of the proximal brachial portion of the arm. There were no paresthesias of the left upper extremity. The patient denied the use of medication, drugs or food supplements, and denied smoking or excessive alcohol use as well.

On physical examination, maximum pain was elicited on active flexion and pronation of the lower arm. Passive extension and resisted flexion of the elbow enhanced the pain on the medial side of the elbow. Movement of the palm and fingers did not increase pain. The biceps and triceps brachii tendons were intact, and the proximal portion of the ulna and the lateral side of the distal upper arm were painful to palpation. There were no neurological or vascular abnormalities of the arm.

**Figure 1:**
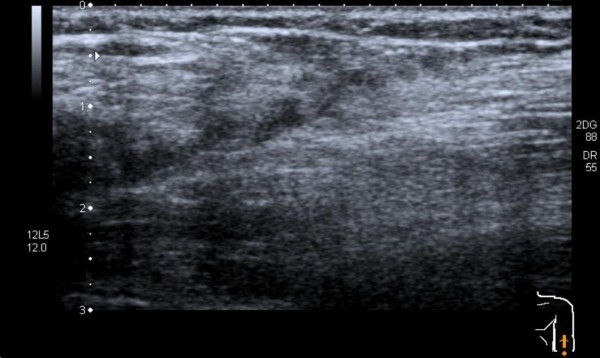
**Inhomogeneous structure with low echogenicity: brachial muscle rupture**.

Conventional radiographs of the elbow revealed no fracture, dislocation or elbow joint effusion. Ultrasound imaging demonstrated an inhomogeneous structure of low echogenicity at the ulnar attachment of the brachialis muscle and direct distally to the coronoid. The brachialis muscle itself revealed another inhomogeneous structure with low echogenicity (Figure 1). The pronator teres muscle was intact.

The diagnosis of a brachialis muscle rupture was made. The affected arm was immobilized for 1 week using a plaster cast. After 1 week, the patient was instructed to gradually exert effort with his arm to maximum tolerable pain. Out-patient follow-up showed a gradual decrease in pain and an improvement in function and strength of the left arm. Near-normal function and strength of the elbow were achieved 10 weeks after the traumatic event.

## Discussion

Injury to the brachialis muscle is a rare phenomenon and is infrequently described in literature [1,2]. This may promote misdiagnosis of this injury. Furthermore, there are conflicting thoughts on the anatomy and the precise function of the brachialis muscle. Gray's Anatomy describes a normal variant with two or more parts [3], while Leonello et al. suggest that all brachialis muscles consist of a superficial and a deep head [4]. The rarity of brachialis muscle injury and the conflicting thoughts on the normal morphology and function of the muscle make diagnosing and treating a brachialis muscle injury a real challenge. The first case report, by Van Den Berghe, presented a male who was clinically diagnosed with a tear of the biceps brachii muscle after lifting a heavy object. However, a MRI revealed a tear in the distal aspect of the brachialis muscle. He was treated conservatively in an outpatient setting and regained full function in 10 months [5].

Nishida et al. described two cases in which the patients were referred for evaluation of a possible muscular neoplasm. Both patients complained of pain and a loss of active extension in the elbow 1 week after the injury. MRI showed a brachialis muscle tear, mimicking an intramuscular tumor. Active mobilization was initiated on both patients with eventual full restoration of function after 3 months [6].

The fourth patient, reported by Winblad et al, was diagnosed with a brachialis muscle tear after a hyperextension injury of the elbow. MRI sequencing confirmed the diagnosis. The patient was treated conservatively with full restoration of function [7].

The final case report was published by Wasserstein and involves a hyperextension injury of the elbow, resulting in a brachialis muscle rupture, confirmed by MRI. Their patient was treated non-operatively and regained full function [8].

To summarize, expensive diagnostic modalities, such as MRI, are too often felt to be needed to definitely diagnose brachialis muscle injury. In our hospital, ultrasound is the first modality of choice if additional studies are needed for diagnosing tendon or muscle ruptures In the case of equivocal findings from the ultrasound imaging, a MRI sequencing is done for the definitive diagnosis. In our experience, we believe that most brachialis muscle ruptures can be treated conservatively with early active mobilization.

## Conclusions

To diagnose peripheral muscle ruptures, ultrasound examination can be an adequate, easy to perform and cost-effective alternative for MRI sequencing in visualizing tendomuscular ruptures. Moreover, we believe that most cases of ruptured brachialis muscle can be treated conservatively.

## Consent

Written informed consent was obtained from the patient for publication of this case report and any accompanying images. A copy of the written consent is available for review by the Editor-in-Chief of this journal.

## Competing interests

The authors declare that they have no competing interests.

## Authors' contributions

TS treated the patient and wrote the case report. ME revised the manuscript critically. Both authors read and approved the final manuscript.
